# From the Veins to the Heart: A Rare Cause of Varicose Veins

**DOI:** 10.1155/2015/849408

**Published:** 2015-05-21

**Authors:** Michele Dalla Vestra, Elisabetta Grolla, Luca Bonanni, Vittorio Dorrucci, Fabio Presotto, Fausto Rigo

**Affiliations:** ^1^Angiology Unit, Ospedale dell'Angelo, Via Paccagnella 11, 30174 Venezia-Mestre, Italy; ^2^Department of Cardiology, Ospedale dell'Angelo, Via Paccagnella 11, 30174 Venezia-Mestre, Italy; ^3^Department of Internal Medicine, Ospedale dell'Angelo, Via Paccagnella 11, 30174 Venezia-Mestre, Italy; ^4^Department of Vascular Surgery, Ospedale dell'Angelo, Via Paccagnella 11, 30174 Venezia-Mestre, Italy

## Abstract

The presence of pulsating varicous veins is an uncommon finding, generically attributed to right heart failure. The precise causes of this phenomenon have been poorly defined in the literature. The finding of this infrequent condition is important because it may be a sign of major diseases, often not known. Here we described a 75-year-old woman presented to the Angiology Unit for the presence of bilateral pulsatile swelling in her groin and along both lower limbs. A bedside ultrasound examination showed an arterial like pulsating flow both in the superficial and in the deep veins of the lower limbs due to a severe tricuspid regurgitation not previously known.

## 1. Introduction

Pulsed Doppler ultrasonographic examination of veins in proximity to the heart shows, in normal condition, a multiphasic wave with two anterograde components, one large systolic wave (S) and one smaller diastolic wave (D), as well as two retrograde waves (a and v). The S wave represents the maximum systolic velocity and is caused by the negative pressure from atrioventricular septal movement toward the cardiac apex. The v wave is produced by positive intra-atrial pressure owing to atrial filling. D wave is consequence of negative intra-atrial pressure resulting from opening of the tricuspid valve. The a wave is produced by the positive intra-atrial pressure, secondary to atrial contraction. This typical waveform is less evident in lower limb veins because the high compliance and high capacitance characteristics of lower extremity venous system dampen the pulsatility and the flow in these venous districts is typically described as spontaneous with a respiratory phasicity [1]. In presence of severe tricuspidal regurgitation a pulsatile flow with a retrograde component has been described in the middle suprahepatic vein, but this characteristic flow along the lower limb veins has been rarely described.

## 2. Case Report

Here we described a 75-year-old woman presented to the Angiology Unit for the presence of bilateral pulsatile swelling and pain in her groin and along both lower limbs. Her medical history included arterial hypertension and bilateral varices along both great saphenous veins. Patient was taking an ACE inhibitor.

**Figure 1:**
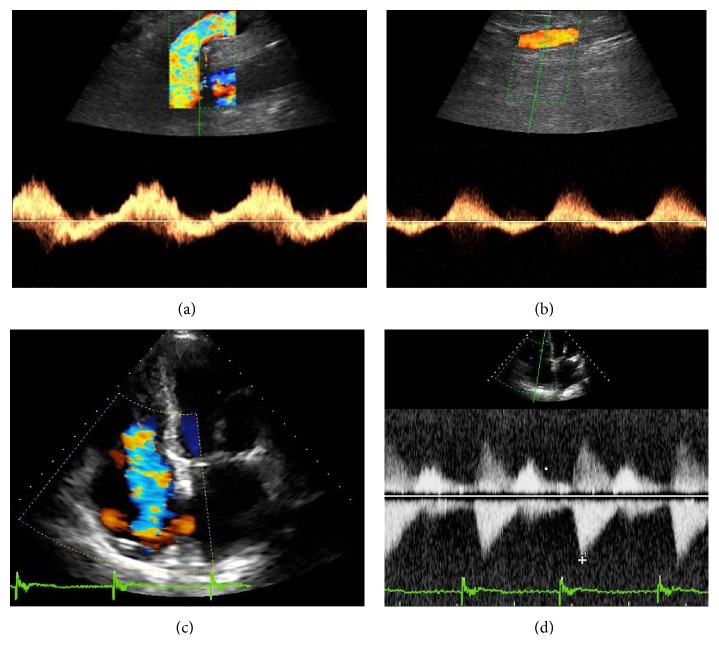
(a) Abnormal pulsatile flow with a retrograde component in saphenous femoral junction. (b) Abnormal pulsatile flow with a retrograde component along the great saphenous vein. (c) Transthoracic echocardiography: severe tricuspid regurgitation at the color Doppler. (d) Transthoracic echocardiography: continuous wave Doppler, showing early peaking and triangular shape of tricuspid regurgitation velocity.

Physical examination confirmed the presence of abnormal bilateral pulsatile groin swelling and pulsating vein ectasias along the entire course of the great saphenous veins. A bedside ultrasound examination was performed. Doppler sonographic examination showed an arterial like pulsating flow in the saphenous femoral junction (Figure 1(a)) and along the great saphenous vein (Figure 1(b)). An arterial like pulsating flow was also observed in the common and superficial femoral veins, in popliteal and tibial veins. This phenomenon was present in both limbs. On the basis of these observations the study was extended to the abdominal and to the jugular veins with evidence of systolic flow reversal. Neither arteriovenous fistula nor an apparent pulsatile flow transmitted by an artery was detected. To better understand this phenomenon a transthoracic echocardiography was also performed. Echocardiography showed the presence of a severe tricuspid regurgitation on color Doppler images (Figure 1(c)), confirmed by early peaking and triangular shape of the velocity at the CW Doppler (Figure 1(d)), increased peak tricuspid E velocity, and systolic flow reversal in the hepatic vein, in presence of annulus dilatation and right ventricular and atrial enlargement. A normal left ventricular ejection fraction (EF 57%) has been recorded. Abnormalities of the aortic and mitral valves were not found.

On the basis of clinical and sonography findings we concluded for severe tricuspid regurgitation with repercussions on the peripheral venous system.

In agreement with the cardiologist diuretics and class 2 graduated compression stokings were prescribed, clinical and cardiovascular imaging follow-up were also programmed.

## 3. Discussion

Varicose veins in lower limbs are common. Most varicose veins are primary; only the minority are secondary to conditions such as deep vein thrombosis and occlusion, pelvic tumours, or arteriovenous fistulae.

In the literature there are some case reports describing pulsating varicose veins secondary to right heart failure in some cases due to tricuspid regurgitation, but the demonstration of an arterial like pulsating flow along such veins is less frequent [2–22].

The lower limb venous Doppler sonography is now widely used by different medical specialists: radiologists, vascular surgeons, internists, cardiologists, and angiologists; the careful interpretation of a simple and widely used venous Doppler ultrasound may open more complex, often not known, scenarios.

In this case report we would like to strengthen the concept that, although infrequent, the presence of pulsating varicose veins and its peculiar waveform could bring out the presence of cardiac abnormalities and direct clinicians to proper course of investigation and management.
